# Polyarticular Septic Arthritis in an Immunocompromised Adult: A Case Report

**DOI:** 10.7759/cureus.104302

**Published:** 2026-02-26

**Authors:** Ana Pais Monteiro, José Nuno Magalhães, Márcia Cravo, Tânia Araújo Ferreira, Miguel Araújo Abreu, Ricardo Sousa, Daniel Soares, Catarina Mendonça

**Affiliations:** 1 Internal Medicine, Unidade Local de Saúde de Santo António, Porto, PRT; 2 Intensive Care Unit, Centro Hospitalar Tâmega e Sousa, Penafiel, PRT; 3 Infectious Diseases, Unidade Local de Saúde de Santo António, Porto, PRT; 4 Orthopaedics, Unidade Local de Saúde de Santo António, Porto, PRT

**Keywords:** immunocompromised infections, mssa invasive disease, polyarticular septic arthritis, rheumatoid arthrits, septic arthrits

## Abstract

Septic arthritis is an inflammatory process affecting one or more joints due to infection, most commonly of bacterial origin. Polyarticular involvement is a rare but severe form of the disease, frequently associated with preexisting joint pathology, involving an average of 3.5 joints and carrying increased morbidity and mortality. We report the case of a 61-year-old woman with rheumatoid arthritis and long-term immunosuppression who was admitted with invasive methicillin-sensitive *Staphylococcus aureus* infection, complicated by polyarticular septic arthritis involving five major joints (left hip, knees, and shoulders), intramuscular abscess formation, and cervical spondylodiscitis. Arthroscopic washout and debridement of the affected joints were performed, and combination antibiotic therapy with flucloxacillin, doxycycline, and rifampicin was initiated and maintained for a total of three months. The patient showed excellent clinical and laboratory improvement, with full recovery from systemic dysfunction. The clinical course, diagnostic work-up, treatment, and outcome are discussed, emphasizing the diagnostic challenges and therapeutic considerations in immunocompromised hosts.

## Introduction

Septic arthritis is defined as the infection of one or more joints, most frequently of bacterial etiology. Its reported incidence ranges from 2 to 60 cases per 100,000 individuals per year [1], with a higher risk observed in patients with predisposing factors such as preexisting joint disease, prosthetic joints, intravenous drug use, diabetes mellitus, chronic alcoholism, skin ulcers, and prior intra-articular corticosteroid injections [2,3]. Typically, septic arthritis presents with monoarticular disease, but polyarticular involvement, although rare, may also occur. Polyarticular septic arthritis (PASA) accounts for approximately 15% of cases and is more frequent in patients with preexisting joint pathology such as rheumatoid arthritis (RA). As polyarticular septic arthritis (PASA) can mimic an acute arthritis flare, and acute rheumatoid arthritis flares may be triggered by systemic inflammation, patients with RA and confirmed monoarticular septic arthritis who subsequently develop polyarticular inflammation can be misdiagnosed or experience delays in diagnosis. However, since PASA is associated with greater morbidity and mortality, with reported mortality rates reaching 49% [4,5], clinicians must be aware of this potential diagnosis. We present a case of invasive methicillin-sensitive *Staphylococcus aureus* (MSSA) infection with polyarticular involvement in a patient with RA, under low-dose methylprednisolone and methotrexate, whilst highlighting the clinical course, diagnostic approach, and therapeutic management.

## Case presentation

A 61-year-old woman with a history of RA, controlled with low-dose methylprednisolone and methotrexate, presented to the emergency department with a four-day history of incapacitating pain in the left hip and fever. On admission, she was febrile (38.1 °C), and physical examination revealed a severely limited range of motion of the left hip, due to pain upon minimal mobilization, which was warm to the touch. Multiple joint deformities consistent with chronic rheumatoid arthritis were observed, including bilateral ulnar deviation at the metacarpophalangeal (MCP) joints, non-tender swelling of both wrists and MCP joints, and bilateral swan-neck deformities involving the fourth and fifth fingers. Non-tender nodules were also noted over the elbows, along with a limited range of motion in the affected joints. However, no signs of active inflammation were present in other joints other than the left hip. Laboratory analysis demonstrated elevated inflammatory markers (C-reactive protein and leukocytosis with neutrophilia) and normocytic normochromic anemia (Table 1). 

**Table 1:** Laboratory results upon admission and after five days of flucloxacilin. Laboratory analysis upon admission showed elevation of inflammatory markers, persistant after initiation of targeted antibiotic therapy ATB: Antibiotic.

Laboratory findings, unit	Admission	Day 5 of ATB	Reference range
Leukocyte count, x10^3^/μL	15.68	24.32	4.00-11.00
Neutrophils, x10^3^/μL	13.96	19.65	2.00-7.50
Lymphocytes, x10^3^/μL	0.49	1.05	1.50-4.00
Monocytes, x10^3^/μL	0.52	0.78	0.20-0.80
Eosinophils, x10^3^/μL	0.02	0.07	0.04-0.40
Basophils, x10^3^/μL	0.08	0.12	0.02-0.10
Hemoglobin, g/dL	11.5	9.3	13-17
Hematocrit, %	35.6	28.2	36-46
Mean cell hemoglobin, pg	29.2	29.9	27.0-32.0
Mean cell volume, fL	90.4	90.7	83-101
Platelets count, x10^3^/μL	419	437	150-400
Sedimentation rate (1st hour), mm	-	97	0-20
C-reactive protein, mg/L	293.19	253.90	0.0-5.0

**Figure 1:**
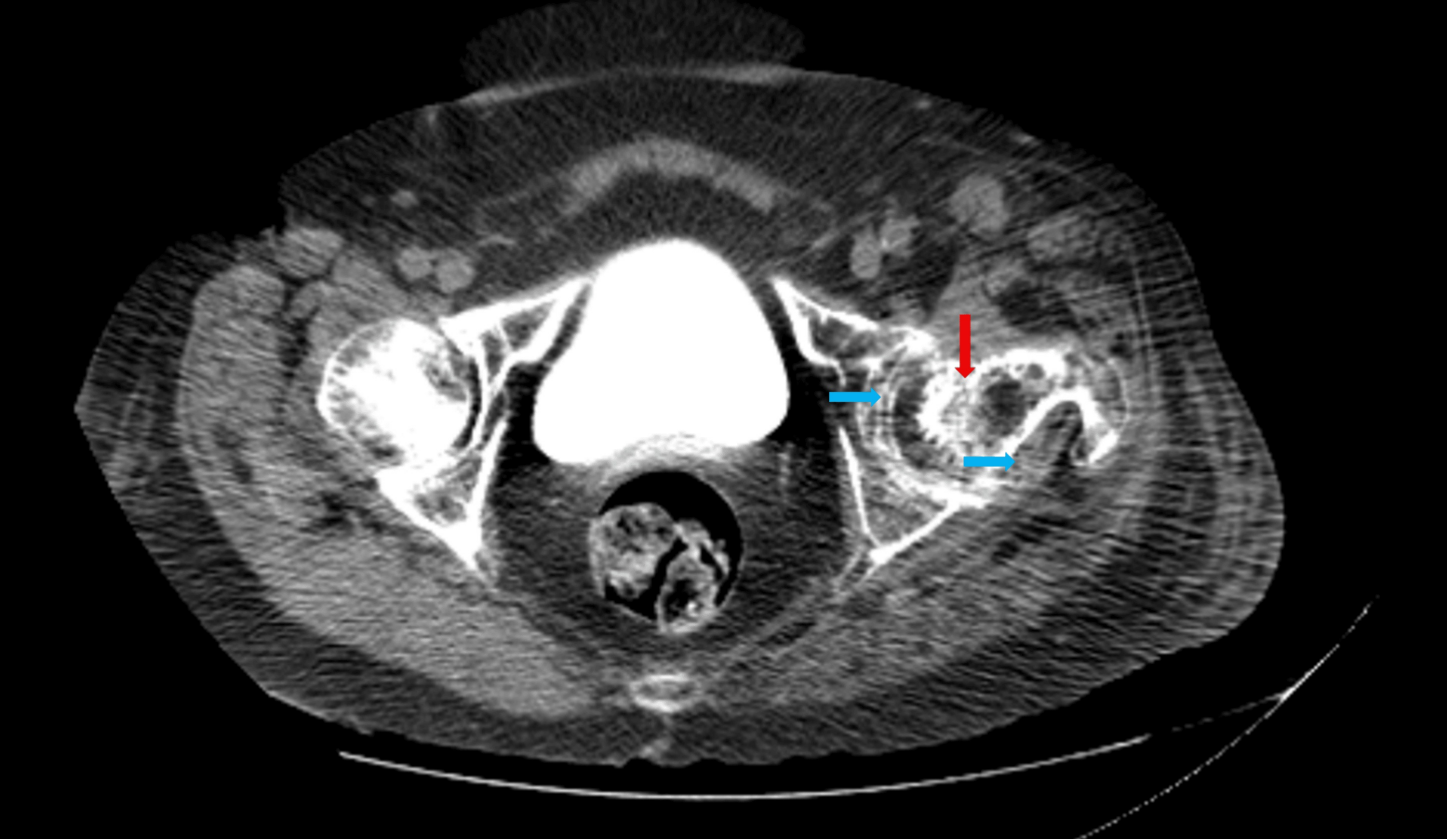
CT scan of the left hip. Extensive destruction and irregularity of the femoral head (vertical, red arrow), accompanied by joint effusion and periarticular soft tissue swelling (horizontal, blue arrows).

Blood cultures demonstrated gram-positive cocci, identified as methicillin-sensitive *Staphylococcus aureus* (MSSA), and antibiotic therapy was thus started with intravenous flucloxacillin. A computed tomography (CT) scan of the left hip showed coxarthrosis with articular effusion, suggestive of active inflammation, and reactive iliac chain lymphadenopathy (Figure 1), raising further suspicion of native joint septic arthritis with extensive femur head destruction. A​​​​​rthrocentesis was performed, and a sample of synovial fluid was sent to microbiological analysis, which showed gram-positive cocci growth, later identified as MSSA, confirming the diagnosis of septic arthritis with bacteremia. 

Methotrexate was discontinued, while low-dose daily prednisolone was maintained to prevent adrenal insufficiency and rheumatoid flare. During the first week of hospitalization, the patient developed bilateral knee pain, with inflammatory signs upon physical examination, initially suggestive of a rheumatoid arthritis flare. However, persistently elevated inflammatory markers and persistent bacteremia raised concern for polyarticular involvement.

Arthrocentesis of both knees yielded purulent synovial fluid, whilst fluid analysis revealed a mild elevation in synovial leukocyte count, predominantly due to polymorphonuclear leukocytes (Table 2). Although leukocyte counts were not markedly elevated, subsequent microbiological cultures isolated methicillin-sensitive *Staphylococcus aureus* (MSSA) from all samples, confirming the diagnosis of polyarticular septic arthritis. 

**Table 2:** Synovial fluid analysis of further joint involvement. Laboratory analysis show slight elevation in synovial white blood cell (WBC) count, mainly due to the presence of polymorphonuclear WBC. Although not significantly elevated, these were performed on day five of targeted antibiotic therapy on a chronically immunosuppressed patient, which can lower the WBC count. Microbiological analysis further revealed Staphylococcus aureus in all samples above.

Synovial liquid, Unit	Left knee	Right knee	Left shoulder	Reference range
Erythrocytes, cells/uL	100	1424	3900	0-0
Leukocytes, cells/μL	2254	12677	30608	0-200
-Polymorphonuclear leukocytes, cells/uL	2116	12293	29185	
-Lymphocytes, cells/uL	46	128	306	
-Monocytes, cells/uL	92	256	1226	
-Other cells, cells/uL	0	0	0	

The patient underwent surgical lavage and debridement of the left hip, with placement of an antibiotic-impregnated spacer, followed by arthroscopic lavage and debridement of both knees, and antibiotic therapy with flucloxacillin was maintained according to culture results and late blood culture sterilization was achieved on the ninth day of treatment. Despite this, a systemic inflammatory state persisted, and new-onset bilateral shoulder inflammation was noted. Suspecting further involvement of these joints, arthrocentesis of both shoulders was attempted. Purulent synovial fluid was encountered in the right shoulder but could not be adequately drained for analysis, while inflammatory synovial fluid was obtained from the left shoulder (Table 2). Cultures from the latter grew MSSA, confirming further joint involvement. Arthroscopic lavage and debridement of both shoulders were subsequently performed, and intraoperative sampling from the right shoulder also yielded the same pathogen. 

**Figure 2:**
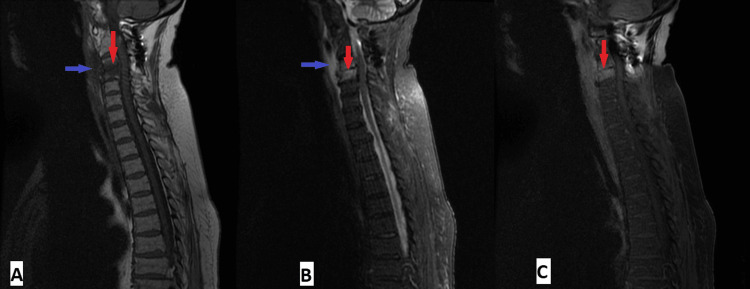
Cervical MRI showing spondylodiscitis. MRI revealed irregularities of the C3-C4 vertebral endplates, accompanied by altered signal from the adjacent bone marrow (vertical red arrows) in both T1 and T2 sequences (A and B, respectively), with enhancement after gadolinium administration (C), reflecting spondylodiscitis in this context, without individualizable lesions/empyema along the vertebral canal, associated with inflammatory signs of the prevertebral muscles, without individualizable abscesses (horizontal, blue arrows).

To exclude other foci, a whole-body CT Scan was performed, which revealed liquefied collections in the iliac and gluteal muscle, as well as spondylodiscitis at the C3-C4 level, confirmed by magnetic resonance imaging, with no myeloradicular compression (Figure 2). Endocarditis was excluded with transesophageal echocardiography. A positron emission tomography (PET) scan did not reveal additional sites suggestive of inflammation. Based on these findings, a diagnosis of invasive MSSA infection with polyarticular involvement, intramuscular abscesses, and C3-C4 spondylodiscitis was established.

**Figure 3:**
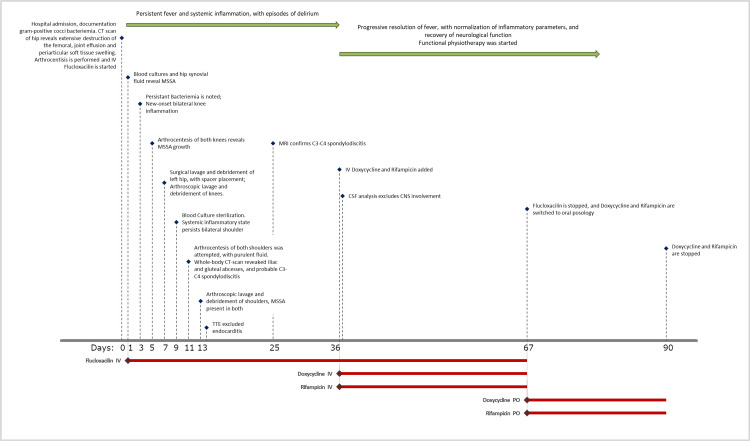
Chronological chart of patient's evolution. TEE: Transesophageal Echocardiogram; CT:Computed Tomography; MRI: Magnetic Ressonance Imaging; IV: Intravenous; PO: *Per os.*

Initial response was slow, and the patient maintained a persistent fever and episodes of delirium, despite appropriate antibiotic coverage. Central nervous system involvement was excluded with cerebrospinal fluid analysis. Given the absence of alternative options and poor clinical response after four weeks of targeted antibiotic therapy, and lack of specific therapeutic guidelines, an enhanced triple antibiotic regimen was initiated, adding doxycycline and rifampicin to ongoing flucloxacillin therapy. This combination was chosen for its synergistic bactericidal effect and enhanced tissue penetration, as well as rifampicin's efficacy against biofilm formation, after spacer placement on the left hip had been performed. No surgical intervention was performed for the intramuscular abscesses or spondylodiscitis, and a conservative, antibiotic-only approach was chosen. The patient responded favorably, achieving defervescence and clinical improvement. This regimen was maintained for two weeks and then adjusted to intravenous flucloxacillin and rifampicin for an additional month, followed by oral doxycycline and rifampicin, completing a total of three months of treatment. The patient’s condition improved steadily, with complete resolution of fever, normalization of inflammatory parameters, and recovery of neurological function (Figure 3).

One month after hospitalization, and approximately two days after initiation of combination antibiotic therapy, the patient sustained a non-traumatic peri-spacer fracture of the left hip, requiring open reduction and spacer replacement. Prolonged infection, preexisting joint damage, chronic corticosteroid therapy, and disuse myopathy were considered contributing factors. Following completion of the antibiotic course, definitive orthopaedic stabilization with a total hip prosthesis was performed, and physical therapy was initiated. The patient resumed ambulating short distances with a walking aid and was discharged to a medium-term rehabilitation unit for physiotherapy and functional recovery. On discharge, therapy for RA was adjusted, and daily hydroxychloroquine was started, maintaining suspension of methotrexate. Evaluation one year after hospitalization showed significant improvement, with no residual pain and ambulatory autonomy with the help of one crutch. 

## Discussion

When presented with* Staphylococcus ​​​​​​aureus *bacteremia with delayed bacteremia clearance, metastatic infection sites should always be suspected; therefore, blood cultures should be taken at intervals of 24-48 hours until proven sterilization [6]. In our case, late bacteremia clearance was present, and when allied with additional joint inflammation, raised suspicion further away from RA flare, and more towards infection seeding. 

Polyarticular septic arthritis is an uncommon but severe clinical entity, most often seen in patients with underlying rheumatologic disease, particularly RA. Other predisposing factors include immunosuppression, malignancy, diabetes mellitus, and chronic kidney or liver disease. It remains unclear whether the increased susceptibility in RA is primarily due to structural joint damage or to the immunosuppressive effects of disease-modifying treatments [3,5,7]. The average number of joints involved in PASA is approximately 3.5, with knees being most frequently affected. In RA patients, the number of joints affected can be underestimated, since PASA can mimic an acute flare of arthritis, posing a diagnostic challenge. However, a delayed diagnosis and inadequate treatment of PASA can lead to irreversible joint destruction, with increased morbidity and mortality, so clinicians should maintain a high index of suspicion to ensure prompt diagnosis and intervention [4,5].

The diagnosis of septic arthritis is mainly based on the synovial leukocyte count of joint fluid, but bacterial identification in synovial fluid remains the most important value. Whilst a high synovial leukocyte (typically, above 50,000 cells/μL) is suggestive of septic arthritis, these cutoffs may not be reliable in immunosuppressed patients, based on expert opinion [1]. Furthermore, in our case, antibiotic treatment had already been started, tailored to the specific pathogen, which can significantly increase the risk of a false negative result. 

As with most cases of *Staphylococcus ​​​​​​aureus *bacteremia, PASA is often associated with concurrent infections at other sites, most commonly endocarditis, soft tissue and airway infections, as well as seeding of implantable medical devices [1,4,5,6]. Compared with monoarticular septic arthritis, PASA carries a worse prognosis, with mortality rates approaching 49% in some studies, mainly when adequate therapy is not employed. Hence, prompt drainage of purulent material and early targeted antibiotic therapy according to the suspected pathogen are crucial to optimize outcomes [2,3,8]. 

There are no standardized guidelines for antibiotic regimens in PASA. The initial choice of antibiotic should be guided by synovial fluid Gram stain, targeting pathogens relevant to the region and local resistance patterns. Relevant data is scarce to support the timing and duration of therapy. The most commonly isolated pathogen is *Staphylococcus aureus*, followed by* Streptococcus spp*.; therefore, the initial antibiotic choice was tailored to microbiological findings. Treatment must be individualized, usually involving a prolonged antibiotic course and should take into consideration other infection sites. In the present case, rifampicin was selected for its bactericidal activity and greater anti-biofilm properties, particularly against staphylococcal infections, when in an appropriate combination regimen such as with doxycycline or flucloxacillin [1,9,10,11]. There is limited current evidence on the surgical approach in the management of septic arthritis, and whilst arthroscopic debridement is an adequate option as initial surgical management in most cases, open debridement could be considered when more structural damage is evident. Furthermore, early joint mobilization, after control of infection, is advised, as to avoid muscular atrophy and contractures [1].

## Conclusions

Polyarticular septic arthritis is a rare but life-threatening condition that requires early recognition and aggressive management. Its occurrence in patients with RA is associated with higher morbidity, mortality, and a more complicated course. Prompt diagnosis, adequate joint drainage, and tailored antibiotic therapy based on microbial culture and patient characteristics are critical for optimal outcomes. A multidisciplinary approach, as illustrated in our case, can further improve patient care by enabling timely diagnosis and successful treatment planning. Clinicians must remain vigilant in differentiating PASA from inflammatory flares in RA, as timely intervention can significantly improve prognosis.
